# Social information and personal interests modulate neural activity during economic decision-making

**DOI:** 10.3389/fnhum.2014.00031

**Published:** 2014-02-06

**Authors:** Anna Moser, Celia Gaertig, María Ruz

**Affiliations:** Department of Experimental Psychology, Mind, Brain and Behavior Research Center, University of GranadaGranada, Spain

**Keywords:** positive and negative interpersonal information, economic decision-making, fairness, uncertainty, MFN, P300

## Abstract

In the present study we employed electrophysiological recordings to investigate the levels of processing at which positive and negative descriptions of other people bias social decision-making in a game in which participants accepted or rejected economic offers. Besides social information, we manipulated the fairness of the assets distribution, whether offers were advantageous or not for the participant and the uncertainty of the game context. Results show that a negative description of the interaction partner enhanced the medial frontal negativity (MFN) in an additive manner with fairness evaluations. The description of the partner interacted with personal benefit considerations, showing that this positive or negative information only biased the evaluation of offers when they did not favor the participant. P300 amplitudes were enhanced by advantageous offers, suggesting their heightened motivational significance at later stages of processing. Throughout all stages, neural activity was enhanced with certainty about the personal assignments of the split. These results provide new evidence on the importance of interpersonal information and considerations of self-interests relative to others in decision-making situations.

## Introduction

It is widely acknowledged that human decisions are not purely rational and outcome maximizing (Camerer, 2003) but that other factors, such as emotions or information about other people, are sources of bias in social decision-making (Harlé and Sanfey, 2007; Ruz et al., 2011). The aim of the current study is to shed light on the neural basis of the bias that evaluative information about other people exerts in interpersonal choices and its inter-dependence with fairness and personal benefit considerations.

Economic games are often used to study strategic human decisions in interpersonal contexts. One of them, the Ultimatum Game (UG; Güth et al., 1982), allows for investigation of how people react to unfair situations. In this game, two players share a certain amount of money. One player offers a split, and the other can then decide whether to accept or reject it. If she/he accepts, both players get the assigned part, whereas if she/he rejects it neither one gets any pay-off. Although the rational decision would be to accept any offer, since a small part of the split is still better than nothing, empirical results show that people reject about 50% of the unfair offers (Camerer, 2003). A prominent explanation of those results refers to the influence of emotions on decision-making (e.g., Pillutla and Murnighan, 1996). Unfair offers elicit negative emotions towards the proposer and trigger punishment of such antisocial behavior; they also activate brain regions linked to emotion processing (Sanfey et al., 2003) and social norm violation (Güroğlu et al., 2010).

Results from Ruz and Tudela (2011) and Ruz et al. (2013) suggest that the congruency of emotions and behavior of interaction partners exerts influence on the decisions we make in a social context. Further, Harlé and Sanfey (2007) found that even incidentally felt emotional states can influence decisions in economic bargaining situations. Social information about the person we interact with can also influence choices made in the UG. In a series of experiments, Ruz et al. (2011; see also Gaertig et al., 2012) showed that verbal descriptions of the personality of interaction partners influence the decisions in a modified UG. More precisely, positive descriptions of alleged partners were shown to increase the acceptance rates of both fair and unfair offers, and this effect was mainly present in an uncertain context in which people did not know the distribution of the offer between the two players.

With electroencephalography (EEG) it is possible to track changes in cortical activation with very high temporal resolution. Therefore, this method gives the opportunity to investigate the stages of processing at which biasing factors exert their influence. The medial frontal negativity (MFN), a specific event related potential (ERP), is of particular interest. This potential summarizes a family of ERPs that are thought to reflect reward prediction errors (the Error Related Negativity) and performance feedback (Feedback Related Negativity; Van Noordt and Segalowitz, 2012). The MFN is thought to originate in the anterior cingulate cortex, a region associated with cognitive control (Carter et al., 1998), negative emotional states (Sanfey et al., 2003) and the processing of both physical and social pain (Rainville et al., 1997). It has been related to performance monitoring and the outcome of decisions (Gehring and Willoughby, 2002), especially the emotional or motivational evaluation of negative outcomes, such as monetary losses (Hajcak et al., 2005). One interpretation of the MFN therefore is that it reflects the evaluation or appraisal of an outcome on a good-bad dimension (Yeung and Sanfey, 2004).

In electrophysiological studies of fairness perception in the UG, Boksem and De Cremer (2010; see also Polezzi et al., 2008; Hewig et al., 2011; Van der Veen and Sahibdin, 2011) found that the presentation of unfair offers elicited a more negative-going MFN compared to the presentation of fair offers. These results suggest that the MFN reflects a fast initial distinction as to whether outcomes adhere to an equity norm. The MFN has been found to also encode other social factors, such as the relationship between the interacting individuals (Ma et al., 2011) or inter-individual differences in social traits (Pfabigan et al., 2011). Campanhã et al. (2011) found that the MFN is strongly responsive to social distance. Crucially, when the economic offer is believed to come from a friend, the polarity of the MFN is reversed, and acceptance rates for unfair offers increase. The authors follow that unfair offers coming from a friend are perceived as less unfair and generate less “social pain” than offers from strangers. This suggests a strong association between the MFN and the ultimate appraisal of an outcome. The authors note, however, that friendship involves much more than social closeness, but also elements like similarity, sympathy and trustfulness. Further, the impact that negative social information might have on the perception of offer fairness remains unclear.

Therefore, a main interest of the present study was investigating whether positive and negative information about otherwise unknown partners modulated the evaluation of the fairness of their offers as reflected on the MFN potential or whether these effects exerted an additive influence. In addition, we manipulated other factors that are also known to affect choices in interpersonal situations. The introduction of advantageous and disadvantageous offers in which the participant either gets the higher or the lower part of the split allows for differentiation between effects elicited by offer *fairness* and those elicited by offer *advantageousness*. When disentangled, the rejection of unfair offers independent of advantageousness reflects an aversion to a violation of an impersonal equity rule (Fehr and Schmidt, 1999). Effects of advantageousness refer to the satisfaction of personal interests in a social comparative context instead. Finally, given that in real life we seldom know all the consequences of our decisions, we also manipulated the uncertainty of the context. Previous results suggest that in an uncertain context, in which participants lack full knowledge about the assignments of the split, social information has a higher impact on choices (Platt and Huettel, 2008; Ruz et al., 2011; Gaertig et al., 2012). The use of electrophysiological recordings allowed us to explore whether social information received enhanced processing in the uncertain context, as previous behavioral data would predict.

Although the MFN was the central potential of interest in our study, there are other ERP deflections that may provide valuable information regarding the levels of information processing at which social information modulates interpersonal decision-making. The P300 peaks around 300–600 ms on centro-parietal sites. It is understood as representing higher-order cognitive operations like decision-making (Nieuwenhuis et al., 2005), or attentional resource allocation (Donchin and Coles, 1988). In gambling studies it has been related to reward magnitude (Yeung and Sanfey, 2004) and reward valence (Hajcak et al., 2005). Employing the UG, Wu et al. (2012) associated the P300 with increased attention depending on the emotional/motivational significance of an outcome in asset distribution. In our study, analysis of the P300 might shed light on the encoding of personal benefit considerations in the human brain.

The present study was designed to investigate if the perception of fair and unfair offers in a modified UG could be altered by previous knowledge about interaction partners in contexts of varying certainty, extending behavioral results about the influence of social information on choices in the UG (Marchetti et al., 2011; Ruz et al., 2011; Gaertig et al., 2012). The study therefore manipulates the *offer fairness* (fair vs. unfair), the *social information* about the interaction partner (positive vs. negative), the *context certainty* (certain vs. uncertain) and the *advantageousness of the offer* (advantageous vs. disadvantageous). At the behavioral level we predict a replication of previous findings of our group concerning the influence of social information on choices in classic (Gaertig et al., 2012) and modified versions (Ruz et al., 2011) of the UG, showing higher acceptance rates for fair and unfair offers following a positive partner description. At the neural level, we hypothesize that the MFN will be modulated by the social information about the interaction partner. We further hypothesize that the P300 will be enhanced by the advantageous offers, given their enhanced motivational significance.

## Methods

### Participants

Twenty-four students from the University of Granada (14 female, mean age: 22.9, age range: 18–34) participated in the study. All subjects had normal or corrected to normal vision. They signed a consent form approved by the Ethics Committee of the University of Granada and received course credits and a chocolate token in exchange for their participation.

### Task

Participants played a modified game used previously by the authors (Ruz et al., 2011) in which they had to either accept or reject economic offers made by a partner. Participants were told that in each trial their partner, the proposer, received an initial amount of fictional money and split it into two parts, one for each of them. The participant then had to either accept or reject the offer. If she/he accepted it they would both earn their share, whereas if she/he decided to reject the proposer’s offer, none would add money for that trial. To enhance closeness to reality, participants were told that offers used in the experiment were made by participants in previous experiments. In addition, to stress that participants’ decision could not influence the offer on the next trial, they were told that they would play with a different proposer on each trial. To introduce the variable of social information, each proposer was described with a positive or negative adjective before the offer was presented. Additionally, and with the goal of getting participants to pay attention to personal benefits, they were told to try to accumulate more fictional money than all their partners together. Finally, we manipulated the certainty of the context in which choices were made. Participants had either full (certain context) or incomplete (uncertain context) information about the outcome of their decisions (see Ruz et al., 2011).

### Stimuli and procedure

Offers were displayed in the center of the screen as two single-digit numbers (from 1 to 9), one for the proposer and one for the responder, separated by a slash symbol. The two numbers were never the same, and their difference was either 1 (fair offers) or 4 (unfair offers). Half of the offers were advantageous, which means that the participant received the higher part of the split, and the other half disadvantageous, assigning the smaller amount of the split to the participant. Participants responded pressing a button on a keypad with the index and middle fingers of their dominant hand (button assignment was counterbalanced across participants). They were instructed to respond as fast as they could, and that the higher part of the split would be added to the amount of the partner if they did not respond within 1500 ms. This information was given to maximize the effect of the verbal descriptions of the partners and to replicate the paradigm employed in previous behavioral studies (Ruz et al., 2011).

For the trait-valenced descriptions, the same 48 words used in a previous study by the authors (Ruz et al., 2011) were selected from the Spanish translation of the ANEW database (Redondo et al., 2007). Half of the words had a positive valence (7.26 in average) and the other half a negative valence (2.19 in average). Words were matched in number of letters (6.5 in average), arousal ratings (5.67 in average) and frequency of usage (26.4 in average; Kucera and Francis, 1967).

To manipulate the certainty of the context, the task was divided into a certain and an uncertain block. Numbers in one block were displayed in different colors (green vs. blue) and in the other block in different font styles (bold vs. underlined). The assignment of color vs. font style to the certain or uncertain conditions was counterbalanced across participants. In the certain block participants were informed of their color/font style and therefore knew which part of the split corresponded to them, whereas this information was not provided in the uncertain block. Even though the different colors/font styles did not reveal any information to the participant in the uncertain block, they were still used to hold visual input constant across blocks. The order of the certain and uncertain blocks (with 384 trials each, and breaks every 96 trials) was counterbalanced across participants. In total, participants received 768 offers. Each participant saw the same word 16 times, each time associated with a different offer. Participants took approximately 70 min to complete the whole task.

**Figure 1:**
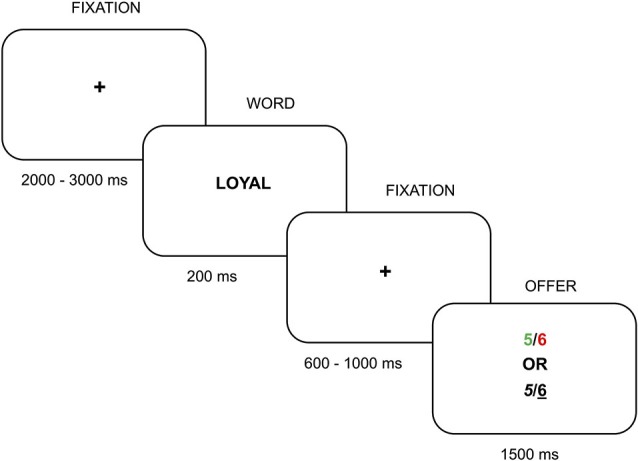
**Schematic display of a trial sequence**.

Each trial comprised a fixation cross (with a variable duration between 2000 and 3000 ms; +; 0.5°), then the positive or negative adjective for 200 ms (average 1.15°), another fixation cross (variable duration between 600 and 1000 ms) and finally the offer for 1500 ms (0.6°; see Figure 1).

### Event related potential (ERP) recording and analysis

An EEG net with 128 electrodes (Geodesics Sensor Net from *Electrical Geodesics, Inc*., EGI) referenced to the vertex channel was used for the electrophysiological recordings. Participants sat in a dimly illuminated and electrically shielded room in front of a 50 cm distant computer screen. After receiving the instructions verbally and in written form, participants first performed a training block to familiarize themselves with the task. To secure good recording quality, participants were instructed to avoid eye-blinking and eye-movements during stimulus presentation. The channels above, beneath and beside the eyes were used as electrooculograph leads to detect eye blinks and movements. Signals were passed through an AC-coupled, high-input impedance amplifier (200 MΩ), and impedance was kept below 50 kΩ for all electrodes. The signal was amplified (0.1–100 Hz band pass), digitized at a sampling rate of 250 Hz (16 bits/D-converter) and stored for off-line analyses.

A 40 Hz lowpass filter was applied to the EEG to remove electrical noise, and afterwards data were segmented into epochs beginning 200 ms before and ending 800 ms after offer onset. Artifact detection was made for eye blinks and saccades (±70 μV threshold) and bad channels (±80 μV threshold). Data were further inspected manually to eliminate remaining bad segments not detected by the software. Channels were replaced with a spherical interpolation algorithm (Pernier et al., 1989) when more than 20% of the trials were bad for a specific channel. Trials with no behavioral response were excluded from the analysis. In order to maintain an acceptable signal-to-noise ratio a criterion of at least 25 trials per condition and subject was established. Data was re-referenced to the average (Tucker et al., 1994), and a single averaged segment was calculated for every condition and subject.

We focused our analyses on 19 anterior-frontal electrodes (number 3, 4, 5, 6, 7, 10, 11, 12, 13, 16, 19, 20, 21, 24, 25, 107, 113, 119, 124) for the MFN and on 16 central-posterior electrodes (number 52, 53, 54, 55, 60, 61, 62, 67, 68, 73, 78, 79, 80, 86, 87, 93) for the P300, where these potentials were maximally distributed. Electrode selection was also aided by localizations reported on previous studies (Boksem and De Cremer, 2010; Wu et al., 2011b). Average amplitudes over these electrodes were calculated with reference to a 200 ms pre-stimulus baseline, with time windows according to those reported in previous literature (Luck, 2005; Boksem and De Cremer, 2010), and also to visual inspection of the timing of waveforms in the present experiment.

For the behavioral results, the choices made by the participants (% of acceptance) were analyzed in a 2 (Context: certain vs. uncertain) × 2 (Fairness: fair vs. unfair) × 2 (Valence: positive vs. negative description of the partner) repeated measures ANOVA. To analyze effects of advantageousness, which refers to personal benefits of an offer compared to the outcome of the partner, data of only the certain block were analyzed in a 2 (Fairness: fair vs. unfair) × 2 (Valence: positive vs. negative description of the partner) × 2 (Advantageousness of the offer: advantageous vs. disadvantageous) repeated measures ANOVA. ERP analyses were performed analogously, submitting the mean amplitudes averaged across channels and temporal windows to the ANOVAs. The Greenhouse-Geisser correction for violations of the assumption of sphericity was used where appropriate and Bonferroni corrections were applied for multiple comparisons.

## Results

### Behavioral results

**Figure 2:**
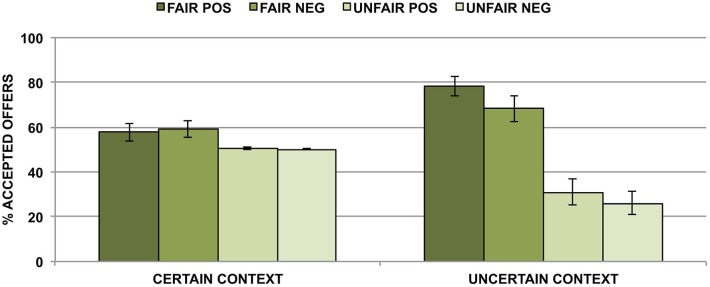
**Acceptance rates for fair and unfair offers following positive and negative descriptions of the interaction partners in certain and uncertain contexts.** Error bars represent standard error of the mean.

Participants responded on time in 97.1% of the trials. The average acceptance rate of the offers was 52.7%. There was a main effect of fairness. Participants accepted more fair (*M* = 65.9%, SE = 3.8%) than unfair (*M* = 39.5%, SE = 2.6%) offers (*F*_1,23_ = 21.20, *p* < 0.001). Valence of the word also had a significant effect on the choice. Participants accepted offers preceded by a positive adjective (*M* = 54.4%, SE = 1.6%) more often than those following a negative adjective (*M* = 51.0%, SE = 1.9%; *F*_1,23_ = 4.30, *p* < 0.05). There was an interaction between the context and the fairness of the offer (*F*_1,23_ = 20.73, *p* < 0.001). The effect of fairness (i.e., acceptance rates of fair minus acceptance rates of unfair offers) was larger in the uncertain (44.7%, *F*_1,23_ = 23.83, *p* < 0.001) than in the certain condition (8.23%, *F*_1,23_ = 4.72, *p* < 0.05). In addition, there was an interaction between the context and the valence of the words (*F*_1,23_ = 6.30, *p* < 0.05). The effect of valence was significant only in the uncertain context (7.5%, *F*_1,23_ = 5.33, *p* < 0.05 vs. *F*_1,23_ = 1.08, *p* = 0.31 in the certain context). There was also a three-way interaction between context, fairness and valence (*F*_1,23_ = 6.47, *p* < 0.05). In both contexts the interaction between fairness and valence was significant (certain: *F*_1,23_ = 5.76, *p* < 0.05; uncertain: *F*_1,23_ = 3.35, *p* < 0.05). In the certain context, acceptance rates of fair offers were marginally higher when preceded by a negative (*M* = 59.35%, SE = 3.82%) than by a positive (*M* = 57.75%, SE = 3.89%) partner description (*F*_1,23_ = 3.94, *p* = 0.06). There was no difference for unfair offers (*F* < 1). In the uncertain condition, acceptance rates of fair offers were higher when preceded by positive (*M* = 78.21%, SE = 4.41%) than by negative (*M* = 68.21%, SE = 5.68%) words (*F*_1,23_ = 5.51, *p* < 0.05). Acceptance rates of unfair offers were marginally higher when preceded by positive (*M* = 30.99%, SE = 5.68%) than by negative (*M* = 26.08%, SE = 5.02%) words (*F*_1,23_ = 3.89, *p* = 0.06; see Figure 2).

The additional analysis yielded a main effect of advantageousness (*F*_1,23_ = 639.6, *p* < 0.001) with higher acceptance rates for advantageous (*M* = 94.6%, SE = 1.5%) than for disadvantageous offers (*M* = 14.0%, SE = 3.2%). An interaction between the advantageousness and the fairness of the offer (*F*_1,23_ = 28.3, *p* < 0.001) showed that when offers were advantageous, unfair offers were accepted more often (97.9%) than fair offers (91.4%; *F* = 5.31, *p* < 0.05). When offers were disadvantageous, fair offers were accepted more often (25.7%) than unfair offers (2.4%; *F* = 15.17, *p* < 0.001). Finally, the effects found in the main analysis were confirmed, showing an effect of fairness (*F*_1,23_ = 4.93, *p* < 0.05) and an interaction between fairness and valence (*F*_1,23_ = 5.76, *p* < 0.05).

### Electrophysiological results

#### Medial frontal negativity (MFN)

**Figure 3:**
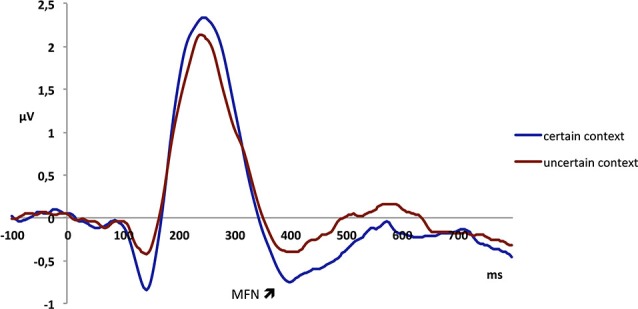
**Electrophysiological data shows that offers presented in the certain context elicit a more negative MFN than those presented in the uncertain context**.

**Figure 4:**
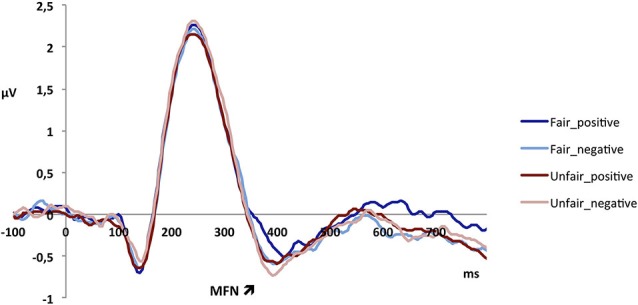
**Electrophysiological data shows that unfair offers elicit a more negative MFN than fair offers, and offers preceded by a negative description of the interaction partner elicit a more negative MFN than those preceded by a positive description.** The effects of fairness and valence of the partner description are additive but do not interact.

**Figure 5:**
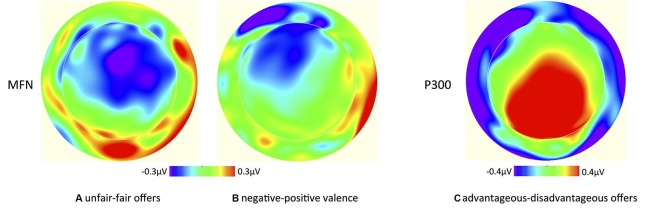
Scalp potential topographies of the average voltage differences between **(A)** unfair and fair offers and **(B)** negative and positive partner description for the MFN, and between **(C)** advantageous and disadvantageous offers for the P300.

The MFN peaked at 385 ms in fronto-central electrodes and was analyzed in a 370–400 ms temporal window. The analysis revealed a main effect of context, with a more pronounced MFN in the certain (−0.61 μV) as compared to the uncertain context (−0.32 μV;* F*_1,23_ = 5.10, *p* < 0.05; see Figure 3). Further, there was a main effect of fairness, as unfair offers elicited a more negative MFN (−0.57 μV) than fair offers (−0.36 μV; *F*_1,23_ = 8.92, *p* < 0.01). There was also a main effect of valence, because a negative description of the proposer elicited a more negative MFN (−0.56 μV) than a positive description (−0.38 μV; *F*_1,23_ = 15.92, *p* = 0.001; see Figure 4; also Figure 5). The interaction between fairness and valence was not significant (*F* < 1).

The additional analysis confirmed the results of the main analysis. A main effect of fairness (*F*_1,23_ = 10.94, *p* < 0.01) and a main effect of valence (*F*_1,23_ = 9.83, *p* < 0.01) were found. Furthermore, there was a significant interaction between the variables valence and advantageousness (*F*_1,23_ = 4.36, *p* < 0.05). Planned contrasts revealed that the effect of valence was only present when preceding disadvantageous offers (negative −0.86 μV vs. positive −0.44 μV; *F*_1,23_ = 12.76, *p* < 0.01), but not when preceding advantageous offers (−0.64 μV vs. −0.58 μV; *F* < 1).

#### P300

**Figure 6:**
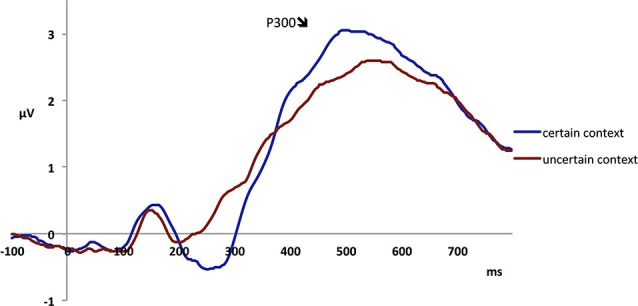
**Electrophysiological data shows that offers presented in the certain context elicit higher P300 amplitudes than those presented in the uncertain context**.

The P300 was analyzed in centro-parietal electrodes in a 370–650 ms time window. The analysis revealed a significant main effect of context, with a higher amplitude in the certain (2.63 μV) than in the uncertain context (2.25 μV; *F*_1,23_ = 4.79, *p* < 0.05; see Figure 6). Further, there was a significant interaction between context and fairness (*F*_1,23_ = 11.43, *p* < 0.01). Planned contrasts showed that in the certain condition, unfair offers elicited a significantly larger P300 than fair offers (2.75 vs. 2.53 μV; *F*_1,23_ = 4.37, *p* < 0.05 ). Conversely, in the uncertain condition, fair offers elicited a marginally significant higher P300 than unfair offers (2.37 vs. 2.15 μV; *F*_1,23_ = 4.00, *p* = 0.06).

**Figure 7:**
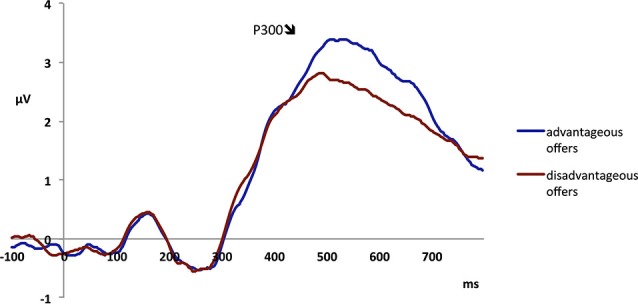
**Electrophysiological data shows that advantageous offers, in which the participant is offered the higher part of the split, elicit higher P300 amplitudes than disadvantageous offers**.

The additional analysis yielded a main effect of fairness (*F*_1,23_ = 4.31, *p* < 0.05), with unfair offers eliciting a higher P300 (2.73 μV) than fair offers (2.51 μV), confirming the effect found in the main analysis. The analysis also revealed a main effect of advantageousness (*F*_1,23_ = 7.73, *p* < 0.05), indicating that advantageous offers elicited a higher P300 (2.83 μV) than disadvantageous offers (2.41 μV; see Figure 7; also Figure 5). There were no other main effects or interactions (all *p*s > 0.05).

Visual inspection of the waveform suggested that there might be separate processes reflected in the early and the late phase of the P300. We therefore performed an additional analysis of an early (370–450 ms) and a late time windows (450–650 ms) of the component. The analyses confirmed the main effect of context (early time window: *F*_1,23_ = 4.41, *p* < 0.05; late time window: *F*_1,23_ = 4.00, *p* = 0.06) and the interaction between context and fairness (early time window: *F*_1,23_ = 4.93, *p* < 0.05; late time window: *F*_1,23_ = 9.24, *p* < 0.01) of the main analysis. However, in the early time window, the effect of advantageousness did not reach significance (*p* > 0.05), whereas this effect was significant in the analysis of the late time window (*F*_1,23_ = 11.70, *p* < 0.01).

## Discussion

The present study was designed to investigate whether social information about other people modulates neural activity at the same neural stages as fairness and personal benefit considerations during interpersonal choices. While EEG was recorded, participants received fair and unfair offers from people previously described either positively or negatively. Both the fairness of the offer and, crucially, the social information about the partner were found to modulate electrophysiological responses, but without interacting between them. Furthermore, the advantageousness of an offer accounted for differential processing of social information about the interaction partner, underscoring the role of personal interest evaluations. Throughout all the stages under study, processing of the offer was increased in the certain as compared to the uncertain context.

At the behavioral level, results were similar to classic findings of the UG (Camerer, 2003), showing that people rejected more than half of the unfair offers. Furthermore, the results confirmed previous findings on the influence of social information on interpersonal choices (Ruz et al., 2011; Gaertig et al., 2012), showing that people accept more offers when these are believed to come from a positively as compared to negatively described person. This shows that non-predictive social information about interaction partners can bias decision-making in interpersonal situations. This behavioral effect was only present in an uncertain context, in which participants lacked complete information about the outcome of their choices. In such uncertainty about the consequences of the decisions, participants seem to make use of each piece of information, independent of its actual validity as a predictor for optimal choice. We also found that the advantageousness of the offer influenced choices and participants accepted more offers when they were assigned the higher amount of the split. This effect interacted with the fairness of the offer, and participants preferred unfair offers when they were assigned the greater part of the split, and fair offers when they were assigned the smaller part of the split. This shows that participants tended to opt for choices which brought them more fictional money than their interaction partners, and, if that was not possible, they preferred offers in which the difference in gains was only small, which is conform to the instructions and also to natural self-interest. A three-way interaction between context, offer fairness and valence indicates that the influence of both fairness and partner description is much more pronounced in the uncertain context. This suggests that when the consequences of an action are less predictable, sources of additional information, such as characteristics of the offer and the interaction partner, have more influence on the decision at hand.

The MFN has been related to the affective appraisal of negative outcomes, such as unfair offers in an UG (Boksem and De Cremer, 2010). Our results replicated the finding of a more negative MFN for unfair offers than for fair ones. Most importantly, the valence of the social information about the interaction partner also had a significant effect on this potential. Negative as compared to positive partner descriptions enhanced the amplitude of the MFN. This effect indicates that offers are evaluated differentially depending on the character of the person that makes the offer. It suggests that as soon as the economic offer is evaluated, it is appraised as a more negative outcome when coming from an unlikable person.

Crucially, we found no interaction between the valence and the fairness of an offer. This indicates that having positive or negative information about the interaction partner does not change the evaluation of the fairness of the offer *per se*. Rather, our results suggest that both fairness and social information add up to generate an overall evaluation of the offer in a positive-negative continuum. This result is in line with the appraisal hypothesis of the MFN (Yeung and Sanfey, 2004), which suggests that the MFN reflects the ultimate appraisal of an outcome.

An alternative theory about the MFN is the Reinforcement Learning approach (Holroyd and Coles, 2002). It refers to expectancy violations and predicts a more pronounced MFN for situations in which previously generated expectations e.g., of fairness are not met. However, our neural results do not support this theory, because in our modified game we find no interaction between the valence of the partner description (which would reasonably inform fairness expectations) and the fairness of the offer. The data therefore rather suggest that the valence of the partner description and the offer fairness independently bias the evaluations of the offer as reflected in the MFN.

Furthermore, our additional analysis including advantageousness in the certain context allowed us to study whether the social information interacted with personal benefit considerations at this stage of processing. In this case, negative social information only enhanced the MFN for offers in which the participant received the lower amount of the split (disadvantageous offers). This effect indicates that social information did not bias the perception of an offer when the sum was split up in a way that privileged the participant. In the condition in which personal interests were satisfied, the personal character of the interaction partner did not seem to have an effect on the affective appraisal of the offer. In contrast, disadvantageous offers from partners described in a negative manner generated a MFN of more negative amplitude than those coming from partners preceded by positive information, which suggests that the offer is appraised more negatively in the former than in the latter case. This result demonstrates the priorities given to the different components of an interpersonal interaction, highlighting in first place personal benefit considerations. It suggests that the character of the interaction partner is considered only when those are not satisfied. When an offer is beneficial, people take less account of the character of the interaction partner.

Another interesting result is that we do not find an interaction between the fairness and the advantageousness of the offer. This suggests that the fairness of the offer modulates the MFN independently of its advantageousness. This is especially interesting because it provides insight into the role of the MFN as a reflection of fairness considerations that are not limited to self-interest. In other studies (e.g., Boksem and De Cremer, 2010) the fairness of the offer was always linked to an advantageous split. Crucially, our design enabled us to distinguish between impersonal offer fairness and personal advantageousness, showing a cleaner effect in the MFN. Our results suggest that the MFN actually reflects an evaluation of fairness, which at this stage of processing is independent of self-benefit considerations. In this line of thought, results of other studies are interesting, showing that witnessing negative outcomes for other people can also elicit MFN responses, which suggest a possible relationship between the MFN and empathy (Thoma and Bellebaum, 2012). It remains a subject for future research to examine the differential MFN depending on outcomes concerning oneself vs. others.

Compared to other reports of this ERP (e.g., Gehring and Willoughby, 2002), the MFN effects found in our study occur relatively late. This might be due to the more complex task design in the current study involving interpersonal information, and/or to the small size of the stimuli employed and lighting conditions (e.g., Wijers et al., 1997). Other studies employing similar tasks (e.g., Wu et al., 2012) tend to also report rather late MFN effects, though not as late as our findings suggest. Yet, it is not fully clear why we find such late MFN effects and future replications are needed to better understand the timing of the component.

At a later stage of processing, the P300 revealed a significant interaction between the context and the fairness of the offer. In the certain condition, where the allocation of the split was disclosed, unfair offers, which are characterized by a greater difference in outcome between both interaction partners, seemed to receive particular attention and elicited a larger P300 than fair offers. The effect was only present in the certain context, which allowed for an outcome comparison with the interaction partner. This suggests that an earlier focus on an impersonal equity rule as reflected in the MFN shifted to social comparative considerations concerning personal interests in the P300. Knowledge about the personal allocation of the split was crucial for enhanced processing of unfair offers, which indicates a role for the P300 in evaluating stimuli relevant to personal interests in a socially comparative setting. In the uncertain condition the effect of offer fairness seemed to be reversed, showing marginally significant higher P300 amplitudes for fair offers. Here, fair offers might have enhanced motivational significance (Yeung and Sanfey, 2004) for the proposer, because even without revealing allocations, these offers do not hold the risk of inequitable treatment. P300 amplitudes were also higher for offers in which the proposer received the higher part of the split (advantageous offers). Evidence showing that the P300 encodes the valence of a stimulus, i.e., win or loss (e.g., Hajcak et al., 2007) suggests that advantageousness in this study could be understood as a social comparative account of stimuli valence. Here, advantageous offers represent an economical benefit in comparison to the gain of the interaction partner. Our P300 results therefore suggest an involvement of the P300 in higher order social cognitive processes (Wu et al., 2011b), in particular social comparison.

It is striking, however, that the effects of fairness and advantageousness in the certain condition were both opposite to results from Wu et al. (2011a, 2012). They found higher P300 amplitudes for equal as compared to unequal splits, as well as for disadvantageous as compared to advantageous unequal splits. The authors interpreted their results by suggesting that participants devoted more attention to disadvantageous offers, because participants might have had to reflect more upon whether to accept or reject such an offer. However, their and our results consistently showed no influence of social information or social distance on the P300 amplitude. This suggests that the early impact of sympathy towards interaction partners was later replaced by strategic or social comparative considerations about outcomes. The separate analysis of early and late time windows of the P300 indicated that the advantageousness of the offer is processed rather late even within the component. This might again reflect the complexity of social comparative considerations in decision-making situations, which leads to a relatively late timing.

Both the MFN and P300 potentials showed a main effect of context, suggesting that throughout all stages of processing, events in the certain context engaged more cognitive resources. Increased amplitudes in the certain condition suggest that attention was drawn to the stimuli that could provide information and that social information was processed in both certain and uncertain conditions, but informed behavioral choices mainly in the uncertain context. Thought to reflect feedback evaluation, the MFN in our experiment was less pronounced in the uncertain condition, where less feedback about outcomes was provided.

There are, however, some limitations in the present study that warrant further investigations. In the first place, the modifications that were made to the UG limit the extension of our findings to the classic game. At the behavioral level, however, our study replicates common findings in the UG. In addition, social information about the partners has also been found to modulate choices in a classic UG setting (Gaertig et al., 2012). Therefore, it would be desirable to replicate our main ERP findings employing a classic UG. Future studies should also improve the ecological validity involving more naturalistic settings and providing a more inherently social environment for the study of interpersonal decision-making, since the current experimental setting has the drawback of artificiality. Previous studies (e.g., Pillutla and Murnighan, 1996; Sanfey et al., 2003) emphasized the role of emotions in decision-making. Regarding the present experiment, it is possible that personal information about interaction partner elicited positive and negative emotions, which might have influenced offer perception and behavioral choices. However, this is only speculative, and further neuroimaging research is needed to determine the active brain regions and associated cognitive processes to integrate the present findings into a bigger picture. Future research could be aimed at exploring how personal interest considerations interact with social information to bias outcome evaluations. Also here, it would be of particular interest to identify the brain regions reflecting such bias. Further, including a neutral condition could help to better understand the impact of positive and negative social information on interpersonal choices.

In summary, our findings underscore the role of social information in interpersonal decision-making situations and show that it affects information processing at several neural stages. As shown, positive and negative character traits of the interaction partner change the appraisal of the offer (MFN potential), in an additive fashion to fairness considerations. Interestingly, whereas such influence is no longer present when economic offers favor personal interests of the responder, personal descriptions modulate valence evaluations of the offers not beneficial to the participant. At a later stage of processing (P300 potential), social comparison mechanisms and personal benefits considerations seem to outweigh influences of the personal characteristics of the interaction partners. Our findings provide new evidence on the importance of social information on the appraisal of outcomes in interpersonal decision situations and its conjoint effects with fairness and personal benefit considerations.

## Author contributions

Conceived and designed the experiment: María Ruz and Anna Moser. Performed the experiment: Anna Moser and Celia Gaertig. Analyzed the data: Anna Moser. Wrote the paper: Anna Moser and María Ruz.

## Conflict of interest statement

The authors declare that the research was conducted in the absence of any commercial or financial relationships that could be construed as a potential conflict of interest.
